# Mesenchymal Stem Cells Accelerate Recovery of Acetic Acid-Induced Chronic Gastric Ulcer by Regulating Ekt/Akt/TRIM29 Axis

**DOI:** 10.1155/2024/6202123

**Published:** 2024-01-03

**Authors:** Feiyue Zhao, Zhibin Fan, Ruikang Jia, Qichao Liu, Menglei Wang, Jianliang Sui, Huiyun Liu

**Affiliations:** ^1^Handan Pharmaceutical Co. Ltd., Handan, Hebei Province, China; ^2^Key Laboratory of Chinese Medicine for Gastric Medicine, Handan, Hebei Province, China; ^3^School of Life Science and Food Engineering, Hebei University of Engineering, Handan, Hebei Province, China

## Abstract

Chronic gastric ulcer (CGU), a prevalent digestive disease, has a high incidence and is seriously harmful to human health. Mesenchymal stem cells (MSCs) have been proven to have beneficial therapeutic effects in many human diseases. Here, a CGU model induced by acetic acid in mice was used to evaluate the repair effects and potential mechanism of human umbilical cord-derived MSCs (hUC-MSCs) and hUC-MSCs derived conditioned medium (hUC-MSC-CM). We found that hUC-MSCs and hUC-MSC-CM treatment significantly repaired morphological characteristics of CGU, improved proliferation and decreased apoptosis of gastric cells, and promoted the generation of new blood vessels in granulation tissues. In addition, we could detect the homing of MSCs in gastric tissue, and MSCs may differentiate into Lgr5-positive cells. As well as this, in vitro experiments showed that hUC-MSC-CM could promote cell proliferation, stimulate cell cycle progression, and reduce the incidence of apoptosis. The transcriptome of cells and the iTRAQ proteome of gastric tissues suggest that MSCs may play a therapeutic role by increasing the expression of TRIM29. Additionally, it was found that knocking down TRIM29 significantly decreased the ameliorative effects of hUC-MSC-CM on cell apoptosis. As a result of further molecular experiments, it was found that TRIM29 is capable of phosphorylating Erk/Akt in specific cell type. As a whole, it appears that hUC-MSCs can be an effective therapeutic approach for promoting gastric ulcer healing and may exert therapeutic effects in the form of paracrine and differentiation into gastric cells.

## 1. Introduction

Chronic gastric ulcer (CGU) is a kind of common digestive disease beyond the muscularis mucosa caused by self-digestion of the gastrointestinal mucosa by gastric digestive juice. There are a variety of clinical symptoms, such as abdominal pain, distension, dyspepsia, acid reflux, and vomiting. According to clinical statistics, the incidence of CGU in the general population is estimated at 5%–10%, which is increasing year by year [1]. CGU is a curable gastric disease, and its clinical treatment is becoming more and more diversified. Some of the most commonly prescribed drugs are omeprazole, lansoprazole, metronidazole, ranitidine, sucralosal, colloidal bismuth, etc. While drug treatment can be effective quickly, there is also the problem of a high recurrence rate after drug withdrawal. Therefore, new and safe therapies, such as the use of stem cells, will be of great benefit to these resistant ulcer patients.

Mesenchymal stem cells (MSCs) are adult stem cells with self-replication potential and multidirectional differentiation. Among the tissues in which MSCs are present are bone marrow, fat, umbilical cord, and placenta. It is possible to obtain MSCs in a wide range of ways. Most tissues of the body can be extracted, such as bone marrow, umbilical cord, and fat. Especially human umbilical cord MSCs (hUC-MSCs), which attract extensive attention due to their advantages of easy access, low cost, and less ethical disputes, are most suitable for scientific research experiments and clinical research. Under specific induction conditions, MSCs can differentiate into bone, cartilage, fat, endothelium, and other cells. MSCs have low immunogenicity and strong immunoregulation. Based on the diversity of the biological characteristics of MSCs, current research on MSCs involves anti-inflammatory treatment, tissue repair and regeneration, graft-versus-host disease (GVHD), autoimmune diseases, and many other aspects, which have broad clinical application prospects [2–5].

MSCs have been used to cure gastric ulcers in several animal models. Once the gastric mucosa is damaged, the gastric mucosa will begin the repair process to restore its original structure and function. This repair mechanism is mainly related to proliferation, apoptosis, cell migration, increased blood flow, and neovascularization of gastric mucosal epithelial cells. For example, several studies have used adipose-derived MSCs (AD-MSCs) to treat animal models of ulcers induced by nonsteroidal anti-inflammatory drugs such as indomethacin [6, 7]. As a result, AD-MSCs could accelerate gastric ulcer healing when injected intraperitoneally in rats, and the submucosal injection of AD-MSCs could facilitate peptic ulcer repair in pigs, respectively. In a study by Xia et al.[8], conditioned medium derived from AD-MSCs was used to treat acetic acid-induced gastric ulcers in rats, and a positive therapeutic effect was observed. To be specific, the secretome from hypoxia-conditioned AD-MSCs could promote the healing of gastric ulcers by enhancing angiogenesis, re-epithelization, and activating the COX2-PGE2 axis through the CCL-20 factor. In another study, bone marrow MSCs (BM-MSCs) were used to assess the antiulcerogenic impact of against gastric ulcers induced by the use of piroxicam in rats [9]. This study suggests that BM-MSCs have the therapeutic ability to cure ulcers because of their high antioxidant activity. Additionally, Liu et al. [10] locally injected AD-MSCs in a mouse model with gastric perforation, which demonstrated that MSCs facilitate the improvement of gastric perforation by an anti-inflammatory process, enhanced cell proliferation, and earlier onset of granulation.

In the current research, we successfully isolated hUC-MSCs using a serum-free culture system, collected hUC-MSCs derived conditioned medium (hUC-MSC-CM), and applied them to acetic acid gastric ulcer mice to explore the therapeutic effect and potential mechanisms of high-quality MSCs on CGU.

## 2. Materials and Methods

### 2.1. Cell Culture

The umbilical cord tissues of full-term newborns were collected in sterile sampling bags with umbilical cord preservation solution (Tianjin Haoyang Biological Manufacture Co., Ltd., TBD2012UCP), then washed with precooled Dulbecco's phosphate-buffered saline (DPBS). Gently removed umbilical arteries, veins, and amniotic epithelium. The remaining umbilical cord tissues were cut into small pieces and washed again with DPBS. The tissues were cultured with ncMission hMSC medium (Nuwacell Biotechnology, RP02010) at 5% CO_2_ atmosphere under 37°C until they reached confluence. We collected a total of five umbilical cord samples and obtained approval from the Ethics Committee of Handan Shengji Cancer Hospital (Handan, China). We mixed and cultivated these five hUC-MSCs at Passage 2. Then, hUC-MSCs at Passages 3–4 were used to follow animal and cell experiments. Osteogenic, adipogenic, and chondrogenic differentiation was induced by a specific differentiation medium (Stemcell).

Human gastric epithelial cells (GES-1) and human gastric cancer cells (HGC27) were cultured in 1,640 medium supplemented with 10% (vol/vol) FBS in a 5% carbon dioxide atmosphere incubator at 37°C.

### 2.2. Conditioned Medium Collection

When the fusion of hUC-MSCs reached 80%–90%, the complete medium was replaced with serum-free F12/DMEM medium, and then the supernatant was collected after 48 hr of culture. Cell debris was removed by centrifugation, and ultrafiltration with a 0.22 *μ*m filter was performed to obtain conditioned medium derived from hUC-MSC (hUC-MSC-CM, abbreviated as MSC-CM subsequently).

### 2.3. Flow Cytometry (FCM) Analysis for MSCs

FCM was used to detect surface markers of hUC-MSCs. The typical positive and negative markers of MSCs were detected by a flow cytometer (BD) using antibodies for CD73 (1 : 40, biolegend), CD90 (1 : 40, biolegend), CD105 (1 : 40, biolegend), CD34 (1 : 40, biolegend), CD45 (1 : 40, biolegend), and human leukocyte antigen DR (HLA-DR) (1 : 40, biolegend), conjugated with PE.

### 2.4. Establishment of the Acetic Acid-Induced Gastric Ulcer Mouse Model

The 6-week-old male Kunming mice (SPF level, SPF (Beijing) Biotechnology Co., Ltd.) were used in this study. We established the mouse model of CGU by acetic acid. The abdominal cavity was opened at the median line of the subxiphoid process, and the stomach was exposed. Filter paper with a diameter of 2 mm was impregnated with 100% acetic acid, and the serosal surface of the junction of the antrum and the body of the stomach was attached twice, 30 s/time. The acetic acid on the surface of the stomach was absorbed with filter paper (the residual acetic acid was rinsed with sterile normal saline), and the left and right sides were performed once (the left side was washed twice for 30 s first and then rewashed with normal saline; the incision was sutured layer by layer, the incision was sterilized with iodophor to protect the incision, and the incision was rewarmed on an electric heating blanket. The mice were put back in the cage and reared in a single cage until the animals woke up. All animal experiments were carried out in accordance with relevant ethical guidelines and regulations and approved by the Experimental Animal Ethics Committee of Kangtai Medical Laboratory Services Hebei Co., Ltd. The approval number of the animal experiments is MDL-2023-09-01-01.

### 2.5. Groups and Tail Vein Injection

We divided the mice into four groups: sham operation group (saline-injected, *n* = 10), model group (saline-injected, *n* = 10), MSC group (MSCs-injected, *n* = 13), and MSC-CM group (MSC-CM-injected, *n* = 10). The second day after the operation, all mice woke up, and a tail vein injection was performed. The MSC group was injected 2 × 10^6^ MSCs through the tail vein. The MSC-CM group was injected with an equal amount of cell-derived conditioned medium through the tail vein. MSC-CM was enriched 50 times using 3 kDa cutoff ultrafiltration tubes (Amicon® Ultra-15, Millipore). The other two groups were injected with the same amount of normal saline through the tail vein. Except for three mice in the MSC group who were sacrificed on Day 15, all the remaining mice were sacrificed on Day 5 (the day of tail vein injection was recorded as Day 0). Blood and stomach samples were collected. Cut along the great bend of the stomach and spread on white paper to measure the surface area of each gastric ulcer with a vernier caliper. Then, rapidly divide each gastric specimen into two halves, one half of which was fixed with 4% paraformaldehyde for HE staining and immunohistochemistry, and the other half was snap frozen in liquid nitrogen and stored at −80°C for immunoblotting.

### 2.6. Hematoxylin and Eosin (H&E) of Gastric Tissue

During the paraffin section, the site of the acetic acid ulcer was taken as the center and sampled along the long axis of the stomach, and the site of the acetic acid ulcer was cut. Slices were scanned using a panoramic slice scanner. According to pathological results, neutrophil infiltration, lymphocytic infiltration, and mucosal edema were scored as reported previously [7, 8]. And the ulcer area, the length of the ruptured muscularis mucosa, and the thickness of the ulcer base were measured as previously described [11, 12].

### 2.7. Immunohistochemistry and TUNEL Staining of Gastric Tissue

For immunohistochemical analyses, sections were incubated with anti-Ki67 (1 : 500, Servicebio), anti-CD31 (1 : 500, Servicebio) and TRIM29 (1 : 100, Santa Cruz). Randomly selected fields (200×) were captured using an upright optical microscope (ECLIPSE E100, Nikon, Japan).

To analyze apoptosis in gastric ulcers, sections were incubated with anti-terminal deoxynucleotidyl transferase dUTP nick end labeling (TUNEL) (Servicebio). Randomly selected fields (200×) were captured using ortho-fluorescent microscopy (ECLIPSE C1, Nikon, Japan).

The Aipathwell software was used to automatically locate the nucleus and expand the cytoplasmic range, and the number and area of positive cells were calculated.

### 2.8. Immunofluorescence

To detect the homing of hUC-MSCs, we used a human nuclear antigen (HNA) antibody (Abcam, ab191181) that only recognizes human cell nuclei for immunofluorescence staining in all groups (Day 5).

Furthermore, we used HNA and Lgr5 (Thermo Scientific, USA) antibodies for dual-color fluorescence staining in the MSC group (Day 15) to detect whether hUC-MSCs can differentiate into target cells.

When 20% MSC-CM was used to treat GES-1 at the specific time, cells were collected for immunofluorescence, and the targets were the Erk1/2 antibody (CST, 1 : 200 dilution) and the phospho-Erk1/2 antibody (CST, 1 : 1,000 dilution). Randomly selected fields (200×) were captured using ortho-fluorescent microscopy (ECLIPSE C1, Nikon, Japan).

### 2.9. Cell Apoptosis

TNF-*α* and SM-164 (Beyotime) were used to induce cell apoptosis in GES-1. GES-1 cells were treated with different concentrations of MSC-CM (0.1%, 0.5%, 1%, 5%, 10%, 20%, and 40%) in combination with TNF-*α* and SM-164. After 24 hr, cells were collected and added with Muse Annexin V& Dead Cell Reagent (Luminex), and apoptosis detection was performed by Muse Cell Analyzer (Luminex).

In addition, the inhibitory effect of 20% MSC-CM coculture on cell apoptosis was detected by TUNEL staining and the expression of caspase3 and poly ADP-ribose polymerase (PARP).

Next, the improvement effect of MSC-CM on apoptosis was detected when TRIM29 was knocked down or not by Muse Cell Analyzer (Luminex). The expression of PARP and TRIM29 was detected by western blot.

All cell experiments were repeated three times.

### 2.10. Cell Proliferation and Cell Cycle

After 24 hr of MSC-CM treatment of GES-1, EdU Cell Promotion Kit and Calcein AM Cell Viability Assay Kit (Beyotime) were used to detect cell proliferation and the number of living cells.

When GES-1 was treated with 20% MSC-CM for 24 hr, the cells were digested and fixed with 70% precooled ethanol. After washing with PBS and degradation of RNA, the PI staining solution was added to fluorescently label the cells, and the cell cycle was detected by FCM (CytoFLEX, Beckman). The cell cycle was analyzed with Modfit software to determine the cell cycle distribution.

All cell experiments were repeated three times.

### 2.11. RNA Sequencing Analysis

RNA-seq technology was used to detect changes in gene expression between control and MSC-CM-treated GES-1. GES-1 of the control group and MSC-CM treated for 24 hr group were collected to extract total cellular RNA (three samples per group). The gene library was obtained using AMPure XP beads, and the library preparations were sequenced on an Illumina Novaseq platform and 150 bp pair-end reads. The final sequencing results were obtained through quality control, comparison, quantification, differential expression gene analysis, and Gene ontology (GO) and Kyoto Encyclopedia of Genes and Genomes (KEGG) enrichment annotation. The corrected *p*-value of 0.05 and the absolute fold change of two were set as the threshold for significantly differential expression. The transcriptome sequencing and analysis were conducted by Novogene Co., Ltd. (Beijing, China).

### 2.12. Proteomic Analysis

For further investigation, iTRAQ technology (isobaric tags for relative and absolute quantitation) was performed to analyze the differential proteins between the Model and MSC groups (six samples per group). A corrected *p*-value of 0.05 and an absolute fold change of 1.2 were set as the threshold for significantly differential proteins. Volcano map analysis, cluster heatmap analysis, and pathway enrichment analysis of GO, KEGG, and InterPro (IPR) were performed for differential proteins. The proteomic sequencing and analysis were conducted by Novogene Co., Ltd. (Beijing, China).

### 2.13. Real-Time Fluorescent Quantitative PCR (qPCR)

MSC-CM treated GES-1 cells were harvested for RNA extraction and reverse-transcribed into cDNA by One-Step gDNA Removal and cDNA Synthesis Supermix (TransGen). TB Green (TaKaRa) was used to detect the expression of the target gene, and then qPCR was carried out on the CFX Connect Real-Time PCR Detection System (Bio-Rad). The primer sequence of the target gene is listed in Table S1. The experiments were repeated three times.

### 2.14. Transfection

In GES-1 and HGC27, empty pcDNA3.1 vectors and TRIM29 plasmids (Sangon Biotech, China) were transfected with DNA transfection reagent (NEOFECT, China) for 24 hr. Control siRNA and TRIM29 siRNA (Sangon Biotech, China) were transfected using Lipofectamine RNAiMAX Reagent (Thermo Scientific, USA) for 24 hr. The sequences for TRIM29 siRNA were designed as follows: sense strand 5′-AGU AGU UGG AGU UCU UGU CGU-3′, antisense strand 5′-GAC AAG AAC UCC AAC UAC UUC-3′ [13].

### 2.15. Western Blotting (WB)

Gastric tissues and cells were collected in a lysis buffer containing protease inhibitors (Beyotime, China). The lysate was homogenized using an ultrasonic processor in such a way that the ultrasound was turned on for 10 s, and the interval was 30 s (FS-150N, SXSONIC, China). Then, centrifuge at 12,000 × *g* at 4°C for 15 min to collect the supernatant. The concentration of total protein was evaluated using the BCA Protein Assay Kit (TIANGEN, China). Erk1/2 antibody (CST, 1 : 2,000 dilution), phospho-Erk1/2 antibody (CST, 1 : 2,000 dilution), Akt antibody (CST, 1 : 2,000 dilution), phospho-Akt antibody (CST, 1 : 2,000 dilution), PARP (CST, 1 : 1,000 dilution), caspase3 (CST, 1 : 1,000 dilution), TRIM29 (Santa Cruz, 1 : 500 dilution), *β*-actin (Proteintech, 1 : 5,000 dilution), and *β*-tubulin antibody (Bioworld, 1 : 5,000 dilution) were utilized. Protein visualization was performed by the Amersham Imager 600 chemiluminescent imaging system (GE Healthcare). The density of western blot bands was quantified by ImageQuant TL software. The experiments were repeated three times.

### 2.16. Statistical Analysis

All data were analyzed using the GraphPad Prism 8.0 software. Results are presented as mean ± SD. Statistical analysis was performed using a one-way analysis of variance followed by Tukey's honestly significant difference test or one sample *t*-test. A value of *P* < 0.05 was considered statistically significant.

## 3. Results

### 3.1. Characterization of Isolated hUC-MSCs

**Figure 1:**
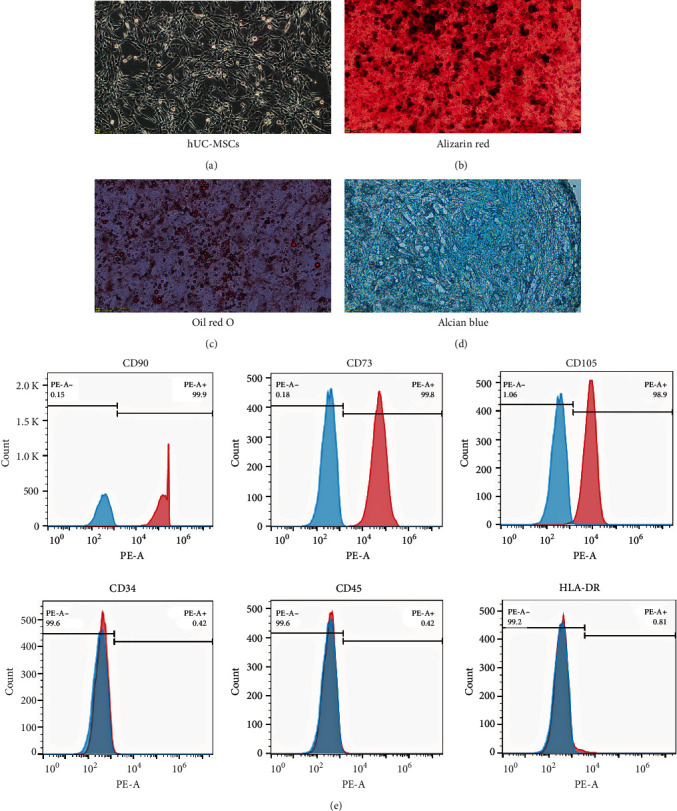
Characterization of isolated hUC-MSCs: (a) morphology of hUC-MSCs under a light microscope (scale bars = 200 *μ*m); (b–d) the differentiation capability of hUC-MSCs was confirmed by Alizarin red S staining for osteogenesis (Scale bars = 200 *μ*m), oil red O staining for adipogenesis (scale bars = 50 *μ*m), and Alcian blue staining for chondrogenesis (scale bars = 50 *μ*m); (e) immunophenotypic characteristics obtained from flow cytometric analysis indicate that the isolated hUC-MSCs expressed mesenchymal markers such as CD73, CD90, and CD105 but negative for hematopoietic lineage markers CD34, CD45, and HLA-DR.

The hUC-MSCs were successfully isolated through the explant method and exhibited a spindle-type morphology during attached proliferation on a culture flask, as shown in Figure 1(a); cell morphology was observed to be similar to that of fibroblasts. To test the multilineage differentiation capacity of the hUC-MSCs, adipogenic, chondrogenic, and osteogenic differentiation kits (Stemcell) were used following the manufacturer's specifications. Finally, osteogenic, adipogenic, and chondrogenic differentiation results were tested through Alizarin Red staining, Oil Red O staining, and Alcian Blue staining, respectively. As shown in Figure 1(b)–1(d), hUC-MSCs could be differentiated into osteogenic, adipogenic, and chondrogenic lineages in vitro. The specific staining for each differentiated cell line was positive. FCM results revealed that hUC-MSCs expressed CD73, CD90, and CD105 positively and CD34, CD45, and HLA-DR negatively, as shown in Figure 1(e). All of these characteristics comply with the criteria defined by the International Society for Cellular Therapy in 2006 (PMID: 16923606). This fully demonstrates that hUC-MSCs isolated and cultured in our laboratory are consistent with MSCs characteristics.

### 3.2. The hUC-MSCs and MSC-CM Promotes Acetic Acid-Induced Gastric Ulcers Healing in Mice

**Figure 2:**
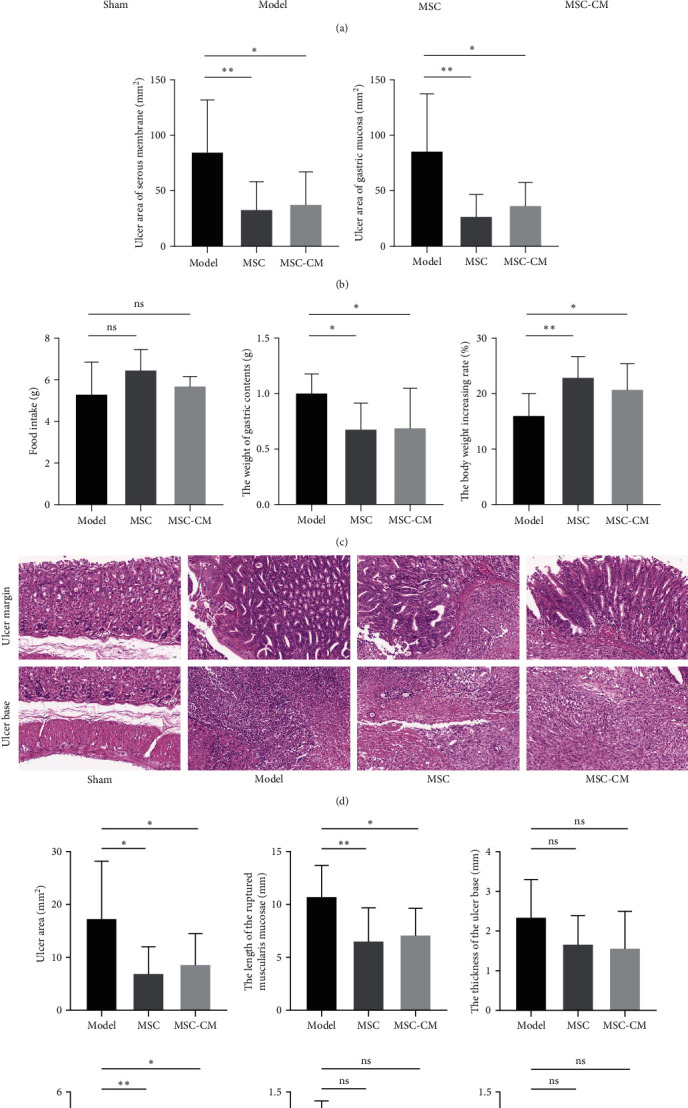
The hUC-MSCs promotes acetic acid-induced gastric ulcers healing in mice: (a) macroscopic observation of gastric ulcers, both serous membrane and gastric mucosa on Day 5 in the groups of Sham, Model, MSC, and MSC-CM; (b) the quantitative results of ulcer area in the gastric serosal and mucosal layers; (c) food take on the Day 4 (the day before the mice were sacrificed), the weight of gastric contents on the Day 5, and the increasing rate of body weight; (d) microscopic images (40×, 200×) of H&E staining of gastric tissues in each group; (e) ulcer area, the length of the ruptured muscularis mucosae and the thickness of the ulcer base of gastric tissues were measured based on H&E staining scanning results. Neutrophils infiltration, lymphocytes infiltration, and mucosal edema of gastric tissues were assessed (*n* = 10) ( ^*∗*^indicate *P* < 0.05,  ^*∗∗*^indicate *P* < 0.01.).

The healing process of CGU was observed by serosa and gastric mucosa, and different responses were observed between the nontreatment and treatment groups. Our preliminary experiment showed that hUC-MSCs and MSC-CM showed a superior effect in healing compared with control. At the Macroscopic observation, the hUC-MSC and MSC-CM treated showed a significantly decreased ulcer area of both serosa and gastric mucosa than the CGU model group at 5 days post-vein tail injection (Figures 2(a) and 2(b)). Compared with the Model group, hUC-MSCs and MSC-CM promote gastric emptying capacity and increase in body-weight at the same ulcer fold intake (Figure 2(c)).

### 3.3. Histopathological Evaluation of Gastric Tissues in Acetic Acid-Induced-Gastric Ulcer

To evaluate the precise effect of hUC-MSCs and MSC-CM, histopathological analysis was taken with Sham, Model, MSC and MSC-CM groups, respectively. Compared with the Model group, H&E staining of MSC and MSC-CM group gastric tissues showed more epithelial regeneration in the mucosal layer of the ulcer margin, decreased neutrophils infiltration, and increased neovasculature in the ulcer area, and improved gastric glandular structure and submucosal connective tissue edema (Figure 2(d)). Additionally, measurement and analysis of H&E staining results showed that the ulcer area and the length of the ruptured muscularis mucosae in the MSC and MSC-CM groups were significantly reduced, which indicated that the gastric mucosa tended to be complete. Due to the short hUC-MSC and MSC-CM treatment period, the thickness of the ulcer base of gastric tissues, lymphocyte infiltration, and mucosal edema of the gastric ulcer area had no significant difference between the treatment group and Model group (Figure 2(e)), which suggested that could take longer to fully recover to normal level. This is consistent with the macroscopic results of hUC-MSC, which demonstrated healing potential in CGU animal models.

### 3.4. The hUC-MSCs and MSC-CM Improved Cell Proliferation and Angiogenesis and Reduced Apoptosis

**Figure 3:**
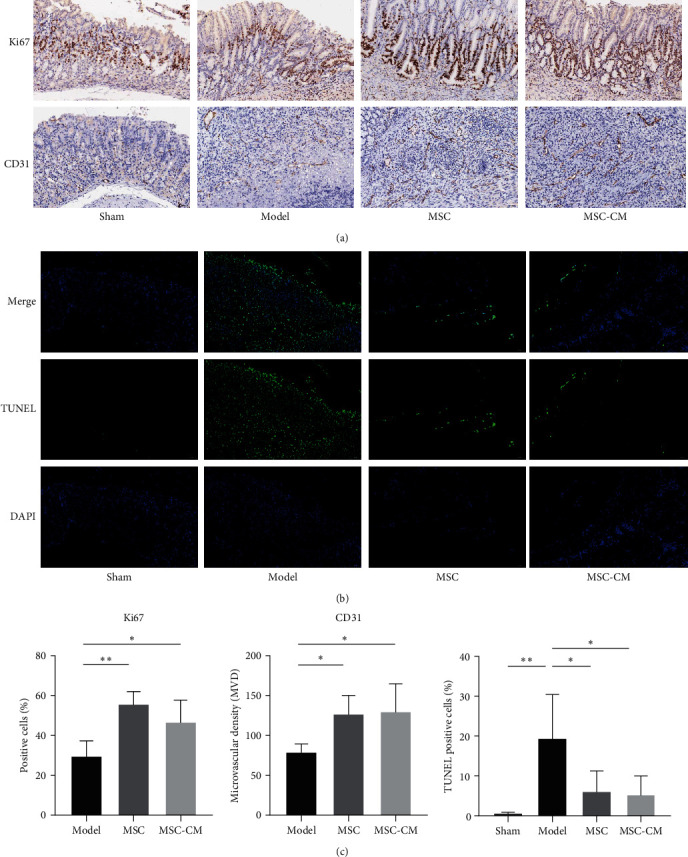
Immunohistochemistry and TUNEL evaluation of gastric tissues in acetic acid induced-gastric ulcer in mice: (a) microscopic images (200×) of Ki67 and CD31 by immunohistochemistry analysis in gastric tissues in each group (*n* = 5); (b) TUNEL staining (scale bars = 100 *μ*m) for assessing apoptosis cells in gastric tissues in each group (*n* = 5); (c) the quantitative results of immunohistochemistry and TUNEL staining.

To verify the repairability for gastric ulcers of hUC-MSCs and MSC-CM, we analyzed cell proliferation and angiogenesis in gastric tissues. We analyzed the positive cell ratio of Ki-67 and microvascular density based on CD31, which were significantly higher in the MSC and MSC-CM groups than in the Model group (Figure 3(a)). A tunnel staining was performed to assess apoptosis in the ulcer area, and green fluorescence signals represented positive cell apoptosis. The results showed an increase in green fluorescence signals throughout the entire ulcer area in the Model group, while apoptotic cells in the MSC and MSC-CM groups were significantly reduced (Figure 3(b)). The quantitative results of Ki-67, CD31, and cell apoptosis are shown in Figure 3(c).

### 3.5. The Presence of hUC-MSCs Could Be Detected in Gastric Tissues of MSC Group

**Figure 4:**
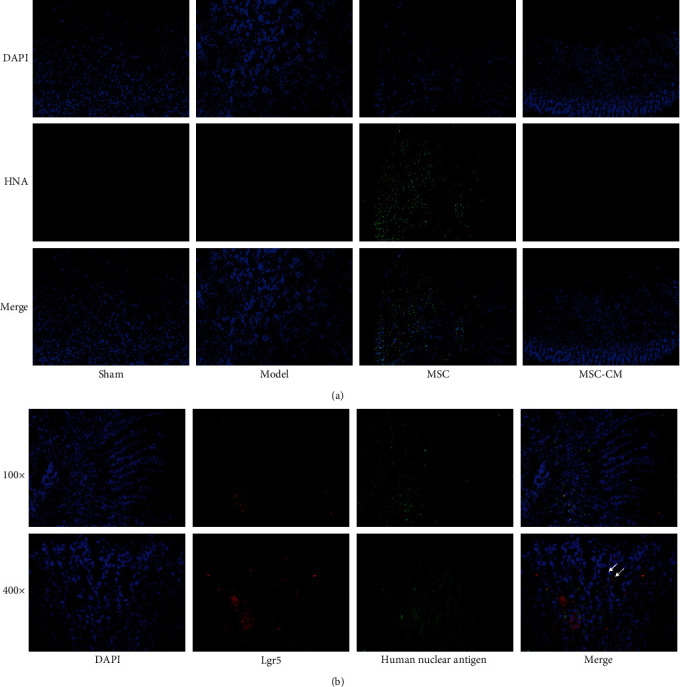
The hUC-MSCs homing detection: (a) the immunofluorescence results of human nuclear antigen (HNA) in the groups of Sham, Model, MSC, and MSC-CM (Day 5); (b) the results of HNA and Lgr5 immunofluorescence double staining in MSC group (Day 15).

We used a HNA antibody to stain gastric tissues of four groups by immunofluorescence. The antibody recognizes only the nucleus of human cells, indicating that cells with a positive green fluorescent signal are hUC-MSCs. No green fluorescence was detected in the other three groups, further confirming the specificity of the antibody (Figure 4(a)). In order to verify whether hUC-MSCs may differentiate into the target cell type, we used HNA and Lgr5 antibodies for immunofluorescence bicolor staining, which showed that a limited number of cells showed dual HNA and Lgr5 positivity, suggesting that after 15 days of tail vein injection, hUC-MSCs may differentiate into Lgr5 positive gastric stem cells in mice (Figure 4(b)).

### 3.6. MSC-CM Significantly Reduced Apoptosis of GES-1 Cells Induced by TNF-*α* and SM-164

**Figure 5:**
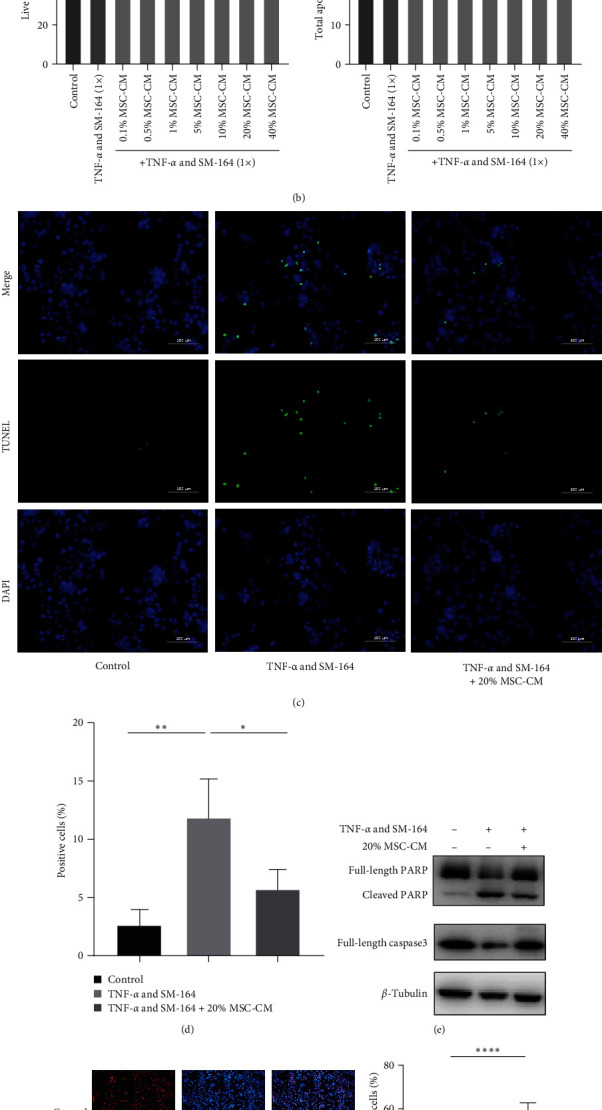
hUC-MSC-CM treatment reduces cell apoptosis and promotes cell proliferation in GES-1: (a, b) the improvement effect of MSC-CM with different concentrations (0.1%, 0.5%, 1%, 5%, 10%, 20%, and 40%) on TNF-*α* and SM-164-induced cell apoptosis in GES-1 cells; (c, d) TUNEL assay was used to detect the effects of MSC-CM on the apoptosis rate of GES-1 cells which induced by TNF-*α* and SM-164, and the percentage of TUNEL-positive nuclei was calculated (scale bars = 100 *μ*m); (e) western blot was used to detect the apoptosis-associated protein caspase3 and PARP in GES-1 cells; (f–i) MSC-CM promotes cell proliferation in GES-1 cells; (j, k) MSC-CM promotes cell cycle progression in GES-1 cells.

We used different concentrations of MSC-CM (0.1%, 0.5%, 1%, 5%, 10%, 20%, and 40%) and cotreated with TNF-*α* and SM-164 to investigate the improvement of MSC-CM on cell apoptosis in GES-1. The results of FCM showed that with the increase of MSC-CM concentration, the number of apoptotic cells significantly decreased, and the number of living cells significantly increased (Figures 5(a) and 5(b)). Therefore, we ultimately chose MSC-CM with a concentration of 20% for subsequent experiments.

We further investigated cell apoptosis using TUNEL staining, which showed that after 20% MSC-CM treatment, the number of TUNEL-positive cells significantly decreased; that is, the number of apoptotic cells decreased (Figures 5(c) and 5(d)).

When GES-1 was treated with TNF-*α* and SM-164 for 24 hr, the full-length PARP decreased, the cleaved PARP increased, and the full-length caspase3 decreased, which fully indicated that the cells underwent apoptosis. However, when 20% MSC-CM was added to coculture for 24 hr, full-length PARP increased, cleaved PARP decreased, and full-length caspase3 increased, indicating that MSC-CM could effectively reduce cell apoptosis (Figure 5(e)).

### 3.7. MSC-CM Promoted the Proliferation of GES-1 Cells

GES-1 cells were shown to proliferate more rapidly during 20% MSC-CM treatment in an Edu cell proliferation experiment (Figure 5(f)). As shown in Figure 5(g), the proportion of proliferating cells increased from 54.67% to 58.50%. As well, after 20% MSC-CM treatment, the number of living GES-1 cells increased from 1,123 to 1,542 by Calcein AM staining at 10× objective (Figures 5(h) and 5(i)). Furthermore, after adding 20% MSC-CM to treat GES-1 cells, the cell cycle of GES-1 changed, and the proportion of S and G2 phase cells increased significantly, indicating that MSC-CM may promote cell proliferation by promoting cell cycle progression (Figures 5(j) and 5(k)).

### 3.8. The hUC-MSCs and MSC-CM Significantly Increased Expression of Erk/Akt/TRIM29 Both In Vivo and In Vitro

**Figure 6:**
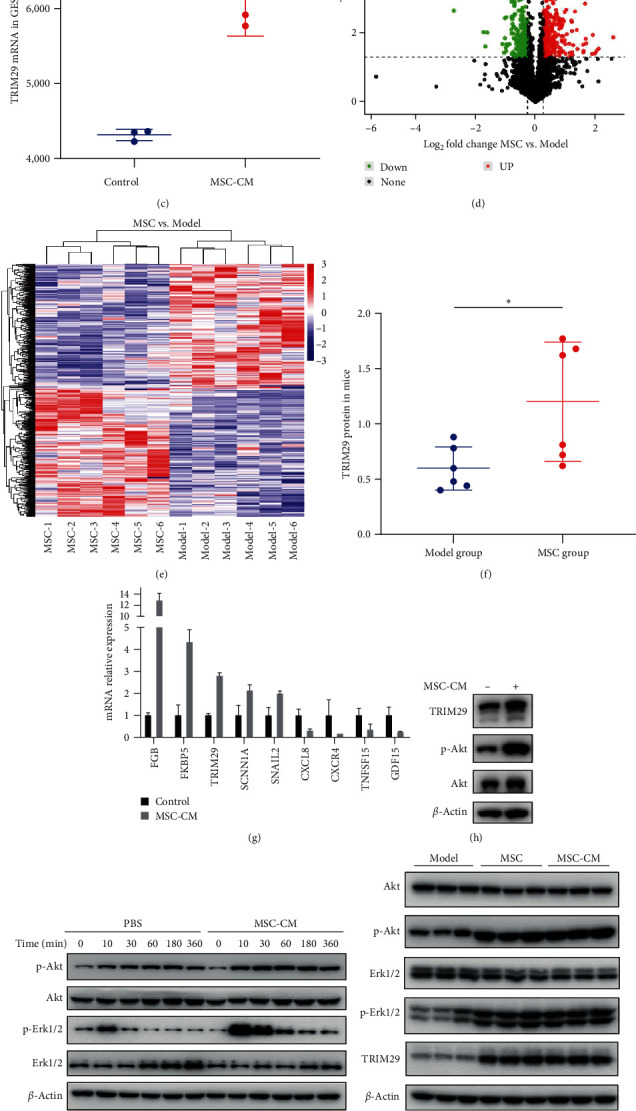
The hUC-MSCs significantly increased expression of Erk/Akt/TRIM29 in vivo and in vitro: (a, b) volcano plots and heat map to determine all differentially expressed genes with a *P*-value of <0.05 between control group and MSC-CM treatment group in GES-1; (c) the TRIM29 mRNA expression in GES-1; (d, e) volcano plots and cluster analysis to show the differentially expressed proteins between the Model and MSC groups in mice; (f) the Trim29 protein expression between the Model and MSC group in mice; (g) verify the changes in related genes by qPCR in GES-1; (h) verify the expression of related proteins by western blot in GES-1; (i) changes in p-Erk1/2 and p-Akt levels in GES-1 cells treated with MSC-CM within 6 hr; (j, k) immunoblots of TRIM29, Erk1/2, p-Erk1/2, Akt, and p-Akt in gastric tissues. The density of western blot bands was quantified by ImageQuant TL software; (l, m) Immunohistochemical and quantitative results of TRIM29 in gastric tissues. The positive cells ratio was quantified by Aipathwell software.

In order to further study the potential mechanism of MSC in treating CGU, we conducted high-throughput sequencing. After treating GES-1 with 20% MSC-CM for 24 hr, the transcriptome was performed, which showed that 80 genes were upregulated and 104 genes downregulated (Figure S1). The volcano map can visually show the distribution of differential genes for each comparison combination (Figure 6(a)). Genes regulated in the upper range are indicated by red dots, and genes regulated in the lower range are indicated by green dots. Genes with similar expression patterns on the heat map are clustered together, with redder colors showing higher expression and greener colors showing lower expression (Figure 6(b)). GO enrichment and KEGG enrichment analysis are displayed in Figure S1. The top 20 significantly upregulated and downregulated genes in GES-1 treated with MSC-CM (fold change > two fold) are listed in Tables S2 and S3, respectively.

At the same time, the proteome of gastric ulcer tissue was performed. Compared to the MSC group and the Model group, the expression levels of 512 proteins changed significantly; 256 proteins were significantly upregulated, and 256 proteins were significantly downregulated. As shown in Figure 6(d), volcano plots show differentially expressed proteins between the Model and MSC groups. Black represents proteins with no significant difference, red represents upregulated proteins, and green represents downregulated proteins.  Figure 6(e) clearly shows the upregulation and downregulation of different proteins when comparing different samples in the form of cluster analysis. GO enrichment, KEGG enrichment, domain enrichment, and interaction analysis are displayed in Figure S2. The 31 significantly upregulated and 12 significantly downregulated proteins (fold change > two fold) in the MSC group are listed in Tables S4 and S5, respectively.

Comprehensive analysis of the transcriptome and proteome showed that MSC could increase the expression of the Trim29 protein, while MSC-CM could increase the expression of TRIM29 mRNA in GES-1. TRIM29 was the only gene that was upregulated at both the cellular and animal levels (Figures 6(c) and 6(f)).

Next, we conducted experimental verification at the cellular and animal levels. qPCR experiment showed that MSC-CM treatment did indeed increase the mRNA expression levels of TRIM29, FGB, FKBP5, SCNN1A, and SNIL2, while reducing the mRNA expression levels of CXCL8, CXCR4, TNFSF15, and GDF15 in GES-1 (Figure 6(g)). Further, western experiments indicated the expression TRIM29 protein was upregulated after MSC-CM treatment for 24 hr (Figure 6(h)). Also, the phosphorylation of Erk1/2 and Akt also increased significantly. The phosphorylation of Erk1/2 reached its highest level after 10 min of MSC-CM treatment, while the phosphorylation of Akt still maintained a high level after 24 hr of MSC-CM treatment (Figures 6(h) and 6(i)). Immunofluorescence experiments showed that after 30 min of MSC-CM treatment, Erk1/2 phosphorylation was higher than that of the control group (Figure S3).

Furthermore, we extracted mouse gastric tissue proteins and conducted immunoblot experiments. The results showed that in the MSC and MSC-CM groups, the expression of Trim29 protein was significantly increased, and the phosphorylation of Erk1/2 and Akt was also significantly increased (Figure 6(j)).  Figure 6(k) shows the quantitative results of the density of protein western blot bands using ImageQuant TL software. Finally, we also performed immunohistochemical testing on gastric tissues. The results showed that in the Sham group, Trim29 was expressed throughout the gastric lamina propria. In the Model group, only a small amount of Trim29 was expressed at the edge of the ulcer, while MSC and MSC-CM groups significantly increased Trim29 expression (Figures 6(l) and 6(m)).

### 3.9. MSC-CM May Activate Erk/Akt Signaling Pathway via TRIM29 Axis

**Figure 7:**
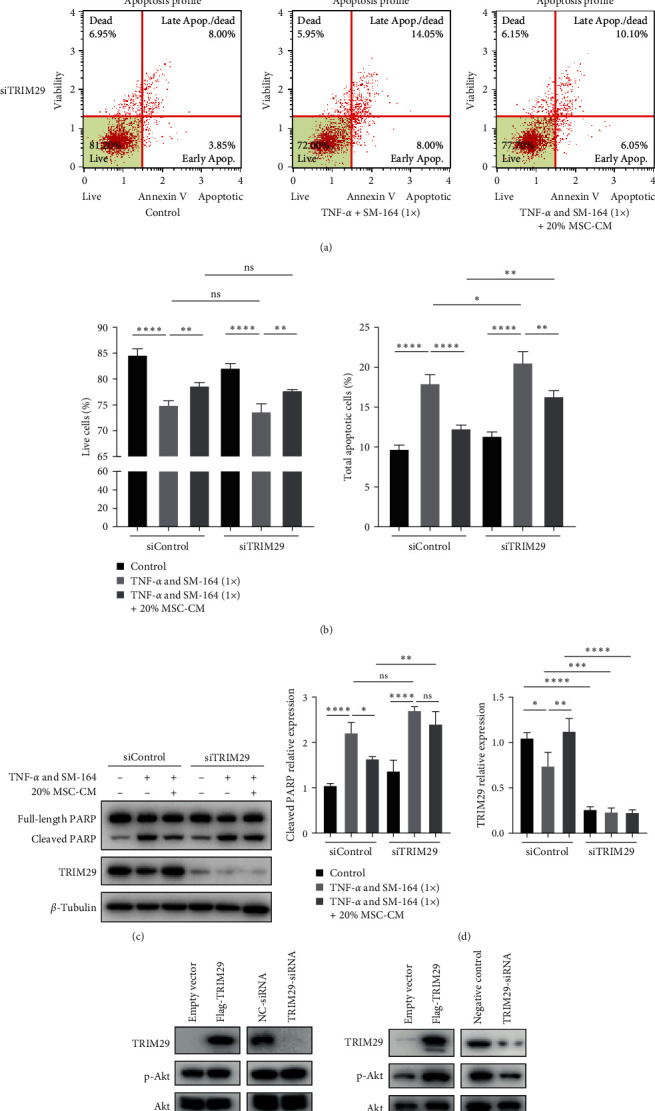
The hUC-MSCs may activate Erk/Akt signaling pathway via TRIM29 axis: (a) knocking down TRIM29 affects MSC-CM in improving cell apoptosis; (b) quantify the number of living cells and the total number of apoptotic cells; (c) knocking down TRIM29, detect the expression level of PARP and TRIM29 protein; (d) the quantitative results of cleaved PARP and TRIM29 protein; (e) overexpression and knockdown of TRIM29 in GES-1 and HGC27, detection of related protein expression.

To further verify the role of TRIM29 in apoptosis, we knocked down TRIM29 in GES-1 for exploration. When control siRNA was added, the number of apoptosis was significantly increased after the addition of TNF-*α* and SM-164, while the addition of MSC-CM could significantly improve apoptosis. After TRIM29 was knocked down, the addition of TNF-*α* and SM-164 increased the number of apoptosis, and even the addition of MSC-CM did not improve apoptosis. In other words, the knockdown of TRIM29 significantly inhibited the effect of MSC-CM on apoptosis (Figures 7(a) and 7(b)). While detecting apoptosis, we also detected the expression of PARP. As shown in Figures 7(c) and 7(d), TRIM29 expression was almost undetectable after the addition of TRIM29 siRNA. The addition of TNF-*α* and SM-164 significantly increased cleaved PARP in the presence of control siRNA, while the addition of MSC-CM reduced cleaved PARP. However, after the addition of TRIM29 siRNA, cleaved PARP increased significantly even in the presence of MSC-CM. These results suggest that MSC-CM may inhibit apoptosis by increasing the expression of TRIM29.

Finally, we overexpressed and knocked down TRIM29 in different cell lines. Surprisingly, it exhibits different situations in different cells. In GES-1 cells, overexpression and knockdown of TRIM29 did not significantly alter the phosphorylation of Erk1/2 and Akt. In HGC27 cells, overexpression of TRIM29 increased Erk1/2 and Akt phosphorylation, whereas knockdown of TRIM29 weakened Erk1/2 and Akt phosphorylation (Figure 7(e)).

## 4. Discussion

CGU is a deep defect in the gastric wall that can cause deep damage to the gastric mucosa and mucosal muscle layer. Gastric ulcers mainly consist of two parts: the ulcer margin and the granulation tissue. The former is an epithelial component of non-necrotic mucosa, while the latter is a connective tissue component composed of fibroblasts, macrophages, and endothelial cells, forming blood vessels and microvessels. The healing of CGU is a complex regenerative process that includes cell proliferation, migration, re-epithelialization, granulation tissue formation, angiogenesis, and various interactions between cells and matrix, all of which are controlled by growth factors, transcription factors, and cytokines [14, 15]. Due to this, it is necessary to explore new clinically applicable therapies for CGU patients who are at risk of recurrence.

Currently, the glacial acetic acid model is widely used in experimental research of the CGU model [8, 11, 12, 16, 17]. Researches show that this model is reliable and reproducible, and the ulcer is deep and large, which is very similar to a chronic human gastric ulcer. This model simulates the occurrence of human gastric ulcers to some extent. Therefore, we used this model to explore the repair effect and mechanism of hUC-MSC on gastric ulcers.

It is particularly important to isolation and culture of umbilical cord MSCs. Good cell status is a prerequisite for the treatment of diseases. Instead of the DMEM/F12-FBS system, we used a commercially available serum-free medium named ncMission hMSC medium (Nuwacell Biotechnology, RP02010) [18–20]. Under the conditions of this culture medium, the cell proliferation ability is very fast and can maintain 10–15 times of proliferation capacity, which ensures that we can obtain a sufficient number of young generations of hUC-MSCs. Considering the heterogeneity of umbilical cords from different sources, we mixed and cultured hUC-MSCs from five different individuals during the second generation. We examined the ability of hUC-MSCs to differentiate into osteogenesis, adipogenesis, and chondrogenesis. We confirmed the high expression of the MSC markers CD73, CD90, and CD105 but not CD34, CD45, or HLA-DR. In subsequent experiments, the third to fourth generations of MSCs were used.

In this study, macroscopic observation showed that the area of gastric ulcer was significantly reduced after MSC and MSC-CM treatment. And further, histopathology showed that MSC and MSC-CM injection through the tail vein could effectively reduce the ulcer area and the length of the ruptured mucosal muscularis and significantly improve gastric mucosa regeneration. In general, MSC and MSC-CM demonstrated healing potential in CGU animal models, both in macroscopic and microscopic observations.

In previous studies, the EGF-R-ERK signaling pathway has been shown to be involved in gastric ulcer healing. A significant increase in Erk1/2 activity was observed in epithelial cells separated from the ulcer edge. Furthermore, Typhostin A46 (an inhibitor of EGF-R kinase and EGF-R kinase-dependent cell proliferation) could delay ulcer healing and reduce EGF-R expression, phosphorylation, and Erk1/2 activity [21]. Multiple studies have fully demonstrated that the phosphorylation of Erk and Akt is accompanied by a significant increase in cell proliferation [22–25]. In our research, we observed a significant increase in phosphorylation of Erk and Akt after MSC and MSC-CM treatment in mice or MSC-CM treatment in GES-1. The high expression of Ki67 in the MSC and MSC-CM group proved that the proliferation of gastric mucosal epithelial cells increased, suggesting that the proliferation of mucosal cells could fill the loss of mucosal tissue caused by injury and then help restore the structure and function of the damaged mucosa. Moreover, MSC-CM treatment remarkably improved the cell proliferation ability in GES-1 and might stimulate cell proliferation by promoting cell cycle progression.

Apoptosis plays an important role in maintaining normal gastrointestinal homeostasis and mucosal integrity [26]. There has been evidence that MSC or MSC-CM can improve cell apoptosis in a variety of disease models [27–30]. We observed fewer TUNEL-positive cells in the MSC and MSC-CM groups; that is, MSC and MSC-CM reduced apoptotic cells in the ulcer area. Additionally, MSC-CM significantly weakened TNF-*α* and SM-164-induced apoptosis in GES-1. These results reveal that reducing apoptosis may be an important factor in the treatment of gastric ulcers.

To explore the impact of MSC-CM on GES-1, we performed transcriptome sequencing. After MSC-CM treatment of GES-1 cells, the expression of the FGB gene was significantly increased. Recent studies have confirmed that overexpression of FGB can significantly promote the tube-formation ability of human brain microvascular endothelial cells (HBMEC), while deletion of the FGB gene can inhibit the tube-forming ability of HBMEC [31]. The high expression of the FGB gene can promote angiogenesis. Another study showed that SIRT1 inhibited renal cell carcinoma (RCC) cell proliferation by inhibiting the expression of the FGB gene, and overexpression of FGB rescued inhibition of SIRT1 overexpression on the proliferation of RCC cells [32]. The expression of the GDF15 gene decreased after treatment with MSC-CM. Studies have shown that GDF15 inhibits tumor growth in the early stage of cancer but promotes tumor cell proliferation in the late stage [33]. Furthermore, GDF15 induced apoptosis in some cancer cells, including A549 and HCT-15, but not others [34, 35]. The effects of GDF15 may be different in different cells [36]. Therefore, we hypothesized that GDF may also play a negative growth regulation role in GES-1. The low expression of GDF15 may enhance the anti-apoptosis ability of cells, which still needs to be confirmed by further studies. In addition, the GO function enrichment analysis enriched differential genes for two pathways, angiogenesis, and epithelial cell proliferation, and related genes include CXCR4, CXCL8, SNAI2, etc.

For further research, we performed proteomics sequencing in gastric tissues. Ly6g6c expression significantly increased in the MSC treatment group. However, there are currently few studies on Ly6g6c. LY6G6C encodes a surface immunoregulatory protein that participates in immune-mediated signal transduction and may be a potential protective biomarker of ulcerative mucositis [37]. Besides, the Ly6 family or its related proteins Slurp2, Ly6d, and Lypd3 are also upregulated in the MSC group. Therefore, we hypothesized that the upregulation of these proteins may play a beneficial role in the healing of gastric ulcers in mice, which, of course, still needs to be confirmed by further studies.

Surprisingly, the keratin family Krt4, Krt1, and Krt6b also show high expression in the MSC group. Keratin is a family of fibrous structural proteins, which are the main proteins that make up the outer layer of hair, horns, claws, and human skin. Keratin protects epithelial cells from damage or stress. *Hericium erinaceus* mycelia could regulate epidermal differentiation by decreasing the expression of Krt16 and Krt6b, thus alleviating ethanol-induced chronic gastric injury in mice [38]. Another study revealed that lotus leaf flavonoids can negatively regulate the expression of Krt16 and Krt6b mRNA in gastric tissues of mice with *Helicobacter pylori* (*H. pylori*)-induced gastric lesions [39]. KrtA and KrtB are overexpressed in skin lesions and epithelial repair and are significantly downregulated in the margin of unhealed venous ulcers [40]. P53 may protect the gastric mucosa from X-ray irradiation by inducing the expression of the keratin family members Krt6, Krt16, and Krt17 [41]. In general, these results suggest that hUC-MSC may contribute to the repair of gastric ulcers by increasing the high expression of the keratin family.

In addition, according to the KEGG enrichment analysis between the Model and MSC groups, autophagy, homologous recombination, and nucleotide excision repair pathways may be involved in the healing of gastric ulcers.

A comprehensive analysis of transcriptome and proteomics sequencing results showed that TRIM29 was up-regulated. In GES-1, MSC-CM treatment increased the expression of the TRIM29 gene by 1.5 times. In mice, the expression of the Trim29 protein in the gastric tissue of the MSC group was twice that of the Model group. The TRIM29 protein is the product of the ataxia telangiectasia group D complementation (ATDC) gene, which has been found to be associated with a variety of biological processes, such as tumor progression and transduction of DNA damage signal. The elevated expression of TRIM29 in colorectal cancer, gastric cancer, and lung cancer leads to an increase in the invasive phenotype of malignant tumors, while in breast cancer and Merkel cell carcinoma plays an inhibitory role [42]. Knockdown of TRIM29 in the gastric cancer cell line MGC803 decreased the expression levels of *β*-catenin, cyclin D1, and c-Myc, indicating that TRIM29 plays a role as an oncogene in gastric cancer [43].

We used WB and immunohistochemistry to confirm that the expression of Trim29 around the ulcer area was decreased in the Model group, but significantly increased in the MSC and MSC-CM groups. To further explore how TRIM29 functions in cells, we performed the knockdown of TRIM29 in GES-1. We found that after TRIM29 knockdown, the addition of TNF-*α* and SM-164 further increased the number of apoptosis compared with that without TRIM29 knockdown, and the number of apoptosis and cleaved PARP were still increased even after MSC-CM was added. These results suggest that MSC-CM could inhibit the occurrence of apoptosis by regulating the expression of TRIM29, which may achieve the effect of treating gastric ulcers.

No reports exist on whether TRIM29 plays a role in the healing of gastric ulcers. However, we speculate that increased expression of TRIM29 improved apoptosis in gastric tissues. Due to the lack of TRIM29-specific inhibitors, we did not validate the role of TRIM29 in gastric ulcers in animals in this study. In subsequent studies, it is necessary to use TRIM29 gene knockout mice to confirm the key role of TRIM29 in the treatment of gastric ulcers.

More and more studies believe that the paracrine effect of MSCs is the main factor in the treatment of various diseases with MSCs. The paracrine effect of MSCs has been confirmed in various diseases, including osteoarthritis, immune diseases, refractory skin defects, GVHD, neurological diseases, liver injury, acute renal failure, and cardiovascular diseases [44–49]. There are also several studies on the paracrine secretion of MSCs in the treatment of gastric ulcers. Researchers found that paracrine effectors released by stem cells play a key role in treating rats and pigs with gastric ulcer models [7, 8]. The exosomes and conditioned medium derived from bone marrow mesenchymal stem cells were used to cure gastric ulcers induced by aspirin in rats. The exosomes restored the tissue structure of gastric mucosa to a more normal state [50].

In this study, MSC-CM showed almost comparable therapeutic effects to MSC at the animal level, suggesting that the paracrine effect of MSCs may be primarily involved in the treatment of gastric ulcers. Nonetheless, MSCs may also differentiate into specific cell types in the stomach to perform their functions; for example, we found that MSCs may differentiate into Lgr5-positive cells. One study has shown that single-cell sequencing of human umbilical cord blood derived MSCs (hUCB-MSCs) transplanted into intrauterine adhesions rabbit models showed that hUCB-MSCs could transdifferentiate into endometrial cells: epithelial cells, fibroblasts, and macrophages, so that the proportion of cells in the damaged endometrial tissues may become increasingly normal [51]. Research has shown that even when gastric ulcers heal visually, regenerative epithelium may exhibit abnormalities for a longer period of time, resulting in abnormal gene profiles of regenerative tissue, insufficient differentiation of gastric cell types, and disrupted cell homeostasis [52]. In future research, we can further explore the specific cell types that MSCs may differentiate in gastric ulcer repair using single-cell sequencing technology, hoping to discover that transplantation of MSCs may result in the cell types being more similar to those seen in normal tissues.

With the continuous improvement of technology, MSCs modified by various genes are gradually being developed. HGF gene-transfected MSCs are used to treat damaged endometrium, hepatocirrhosis, and ameliorate cerebral ischemia/reperfusion injury [28, 53, 54]. Engineered basic fibroblast growth factor-overexpressing MSCs improve evident therapeutic outcomes in mouse spinal cord injury models [55]. CCR2-overexpressing MSCs enhances the targeted migration of MSCs to damaged liver and improves the therapeutic effect of acute liver failure [56]. In the future, in order to treat gastric ulcers more effectively, we can use transgenic overexpression of growth factors or chemokines in MSCs to enhance their therapeutic efficacy and homing ability, leading to earlier ulcer healing.

## 5. Conclusions

In this study, we used umbilical cord MSCs and conditioned medium to treat acetic acid-induced gastric ulcers in a mouse model and found that they can effectively reduce ulcer area, possibly promoting gastric epithelial cell proliferation through the Erk/Akt pathway, effectively reducing cell apoptosis through TRIM29, and promoting ulcer basal angiogenesis. In general, although MSCs may function through paracrine mechanisms, they cannot be excluded from undergoing differentiation into gastric cells. Erk/Akt/TRIM29 axis may be one of the potential targets for MSC to play a therapeutic role in gastric ulcer healing.

## Data Availability

The data presented during the current study are available upon request from the corresponding author on reasonable request.

## Supplementary Materials

Supplementary Materials Figure S1: RNA-sequencing analysis of control and MSC-CM-treated GES-1 cells. Figure S2: proteomic analysis of the Model and MSC group. Figure S3: immunofluorescence of ErK1/2 and p-ErK1/2 in GES-1 treated with DPBS or MSC-CM (20%) at 0 or 30 min. Table S1: the primers were used in qPCR. Table S2: the list of top 20 significantly upregulated genes (fold change > two fold) in MSC-CM treated GES-1. Table S3: the list of top 20 significantly downregulated genes (fold change > two fold) in MSC-CM treated GES-1. Table S4: the all of 31 significantly upregulated proteins (fold change > two fold) in the MSC group. Table S5: the all of 12 significantly downregulated proteins (fold change > two fold) in the MSC group.
