# Supplementary material for: Mesenchymal Stem Cells Accelerate Recovery of Acetic Acid-Induced Chronic Gastric Ulcer by Regulating Ekt/Akt/TRIM29 Axis
Source: Stem Cells Int. 2024 Jan 3;2024:6202123. doi: 10.1155/2024/6202123 (PMC10781525; doi:10.1155/2024/6202123)
Supplement: Supplementary Materials — Figure S1: RNA-sequencing analysis of control and MSC-CM-treated GES-1 cells. Figure S2: proteomic analysis of the Model and MSC group. Figure S3: immunofluorescence of ErK1/2 and p-ErK1/2 in GES-1 treated with DPBS or MSC-CM (20%) at 0 or 30 min. Table S1: the primers were used in qPCR. Table S2: the list of top 20 significantly upregulated genes (fold change > two fold) in MSC-CM treated GES-1. Table S3: the list of top 20 significantly downregulated genes (fold change > two fold) in MSC-CM treated GES-1. Table S4: the all of 31 significantly upregulated proteins (fold change > two fold) in the MSC group. Table S5: the all of 12 significantly downregulated proteins (fold change > two fold) in the MSC group. [file 6202123.f1.docx]

**Supplementary materials**


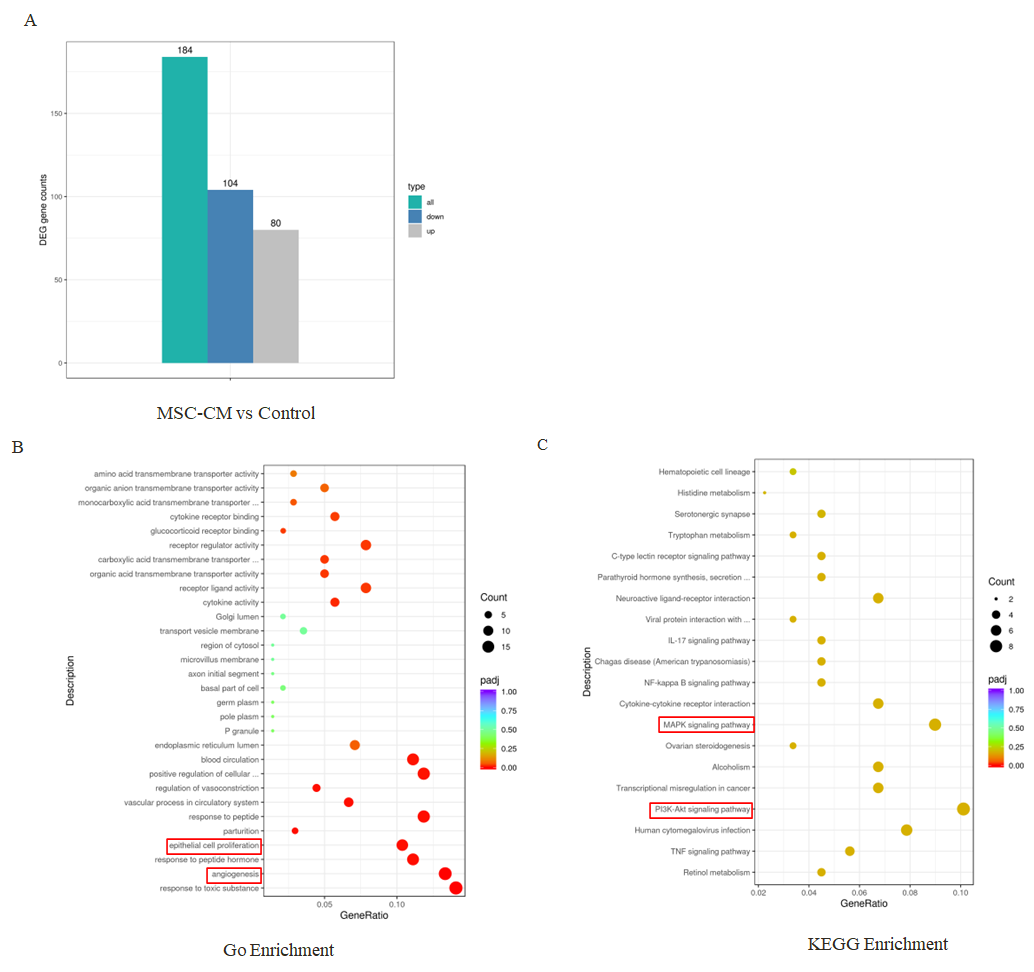
 Figure S1 RNA-sequencing analysis of control and MSC-CM treated GES-1 cells. (A) Number of differentially expressed genes after MSC-CM treatment. (B) GO enrichment analysis of the top 20 pathways between control group and MSC-CM treatment group. (C) KEGG enrichment analysis of the top 20 pathways between control group and MSC-CM treatment group.

Among the pathways through GO enrichment of the top 20 pathways between the control group and the MSC-CM treatment group, two pathways are related to our study, angiogenesis and epithelial cell proliferation (Figure S1B). According to the ratio of the number of differential genes annotated to the KEGG pathway to the total number of differential genes, the top two pathways are PI3K-Akt signaling pathway and MAPK signaling pathway (Figure S1C).


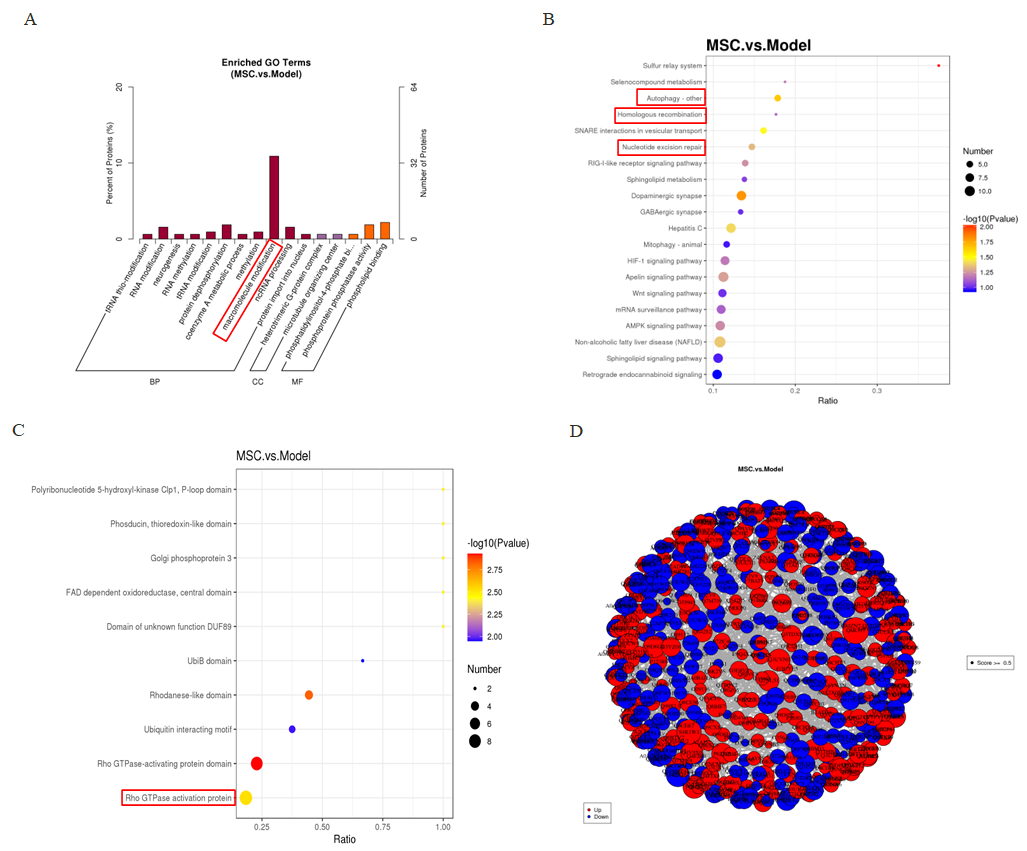


Figure S2 Proteomic analysis of the Model and MSC group. (A) Go enrichment analysis of the all pathways between the Model and MSC group with a p-value of < 0.05. (B) KEGG enrichment analysis of the top 20 pathways between the Model and MSC group. (C) IPR enrichment analysis of the top 10 structural domains between the Model and MSC group. (D) Interaction analysis of identified proteins using StringDB protein interaction database.

The Go enrichment analysis of all 16 pathways between the Model and MSC group with a p-value of < 0.05 was shown in Figure S2A, among which the pathway with the highest number of enriched proteins was macromolecular modification. Among the KEGG enrichment analysis of the top 20 pathways between the Model and MSC group, autophagy, homologous recombination, and nucleotide excision repair pathways may be the most relevant for our study (S2B). Domain enrichment can find domain entries that are statistically significantly enriched. This function or positioning may be the cause of the difference. The domain most statistically significant is the Rho GTPase-activating protein domain (S2C). The StringDB protein interaction database (http://string-db.org/) was used for interaction analysis of identified proteins, and the network diagram was constructed, as shown in Figure 2D.


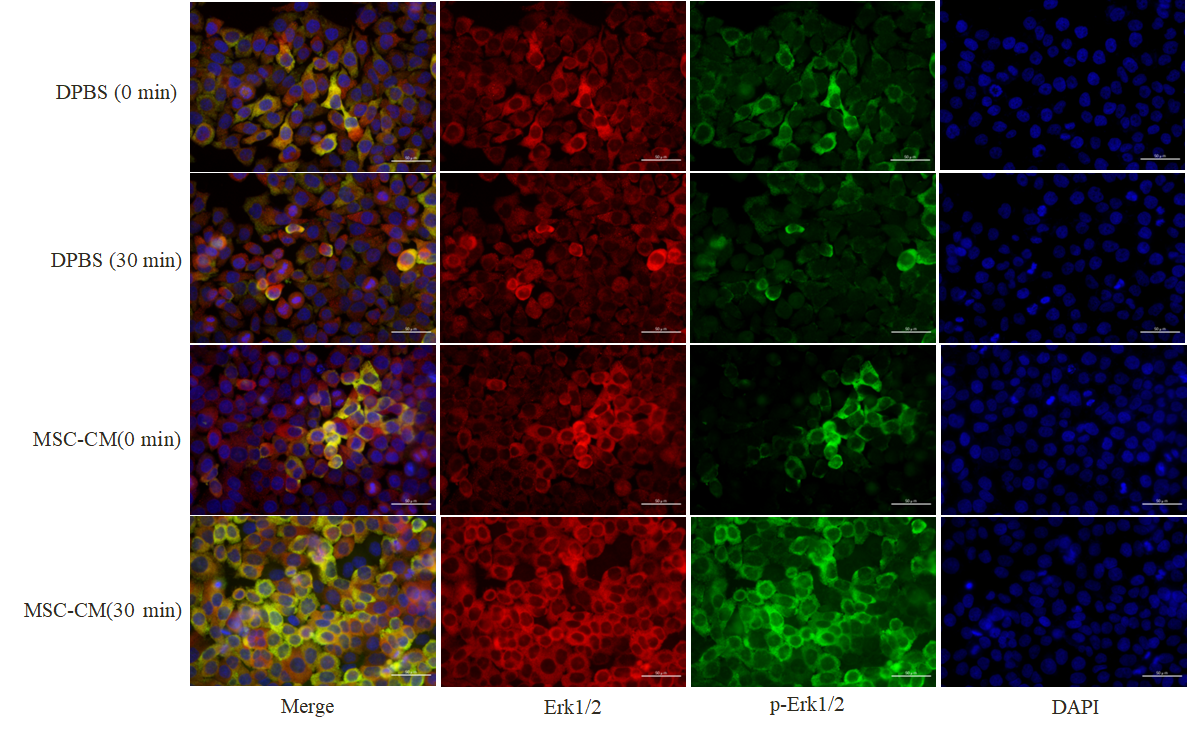


Figure S3 Immunofluorescence of ErK1/2 and p-ErK1/2 in GES-1 treated with DPBS or MSC-CM (20%) at 0 or 30 min.

Table S1 The primers were used in qPCR.

| Primer name | Primer sequence (5’→3’) | Size (bp) | T_m_ (°C) |
| --- | --- | --- | --- |
| β-actin-F | GCCGACAGGATGCAGAAGGAGATCA | 197 | 58 |
| β-actin-R | AAGCATTTGCGGTGGACGATGGA |  |  |
| FGB-F | TGACAGAGACAATGACGGCT | 150 |  |
| FGB-R | TTTGCCATGTCCCAGGTGTA |  |  |
| FKBP5-F | TCTCCTTGCTGCCTTTCTGA | 173 |  |
| FKBP5-R | CACCCTTGGCTGACTCAAAC |  |  |
| TRIM29-F | AAGGAGAAGGACCGCATCAA | 189 |  |
| TRIM29-R | CACCTTGGCTCTCTCATCCA |  |  |
| SCNN1A-F | CCTACCGAGAGCTCTTCGAG | 248 |  |
| SCNN1A-R | ATTGAGGGTGCAGATGGTCA |  |  |
| SNAI2-F | CCTGGTTGCTTCAAGGACAC | 204 |  |
| SNAI2-R | AGCAGCCAGATTCCTCATGT |  |  |
| CXCL8-F | CAGTTTTGCCAAGGAGTGCT | 207 |  |
| CXCL8-R | ACTTCTCCACAACCCTCTGC |  |  |
| CXCR4-F | AACACGAGGATGGCAAGAGA | 223 |  |
| CXCR4-R | GAGTCATAGTCCCCTGAGCC |  |  |
| TNFSF15-F | AGAGCAGACGGAGATAAGCC | 203 |  |
| TNFSF15-R | CGGAATGTGACCTGGGAGTA |  |  |
| GDF15-F | ACGGTGAATGGCTCTCAGAT | 213 |  |
| GDF15-R | GGTGTTCGAATCTTCCCAGC |  |  |

Table S2 The list of top20 significantly up-regulated genes (Fold Change >2 fold) in MSC-CM treated GES-1.

| **Gene _ID** | **Gene name** | **Gene description** | **log2FoldChange** | **Pvalue** |
| --- | --- | --- | --- | --- |
| ENSG00000171564 | FGB | fibrinogen beta chain | 4.4985 | 0.0000 |
| novel.591 | - | - | 4.3568 | 0.0058 |
| ENSG00000111319 | SCNN1A | sodium channel epithelial 1 alpha subunit | 3.3470 | 0.0000 |
| ENSG00000171862 | PTEN | phosphatase and tensin homolog | 2.9510 | 0.0073 |
| ENSG00000127129 | EDN2 | endothelin 2 | 2.9220 | 0.0000 |
| ENSG00000153495 | TEX29 | testis expressed 29 | 2.7597 | 0.0000 |
| ENSG00000069431 | ABCC9 | ATP binding cassette subfamily C member 9 | 2.5105 | 0.0000 |
| ENSG00000169067 | ACTBL2 | actin beta like 2 | 2.3776 | 0.0000 |
| ENSG00000125148 | MT2A | metallothionein 2A | 2.3473 | 0.0000 |
| ENSG00000134830 | C5AR2 | complement component 5a receptor 2 | 2.3307 | 0.0000 |
| ENSG00000189221 | MAOA | monoamine oxidase A | 2.3177 | 0.0000 |
| novel.155 | - | - | 2.2841 | 0.0000 |
| ENSG00000019549 | SNAI2 | snail family transcriptional repressor 2 | 2.1777 | 0.0000 |
| ENSG00000166741 | NNMT | nicotinamide N-methyltransferase | 2.0991 | 0.0000 |
| ENSG00000096060 | FKBP5 | FKBP prolyl isomerase 5 | 2.0479 | 0.0000 |
| ENSG00000116741 | RGS2 | regulator of G protein signaling 2 | 1.9791 | 0.0000 |
| ENSG00000079385 | CEACAM1 | carcinoembryonic antigen related cell adhesion molecule 1 | 1.9627 | 0.0000 |
| ENSG00000226281 | AL031123.1 | novel transcript | 1.9488 | 0.0000 |
| ENSG00000179094 | AC129492.1 | period circadian regulator 1 | 1.8834 | 0.0000 |
| ENSG00000169715 | MT1E | metallothionein 1E | 1.7553 | 0.0000 |

Table S3 The list of top20 significantly down-regulated genes (Fold Change >2 fold) in MSC-CM treated GES-1.

| **Gene _ID** | **Gene name** | **Gene_description** | **log2FoldChange** | **Pvalue** |
| --- | --- | --- | --- | --- |
| novel.530 | - | - | -5.6830 | 0.0001 |
| ENSG00000181634 | TNFSF15 | TNF superfamily member 15 | -2.9001 | 0.0000 |
| ENSG00000130513 | GDF15 | growth differentiation factor 15 | -2.3715 | 0.0000 |
| ENSG00000173391 | OLR1 | oxidized low density lipoprotein receptor 1 | -2.2499 | 0.0000 |
| ENSG00000111181 | SLC6A12 | solute carrier family 6 member 12 | -2.1648 | 0.0000 |
| novel.291 | - | - | -2.1167 | 0.0047 |
| ENSG00000257093 | KIAA1147 | KIAA1147 | -1.9843 | 0.0005 |
| novel.165 | - | - | -1.9511 | 0.0000 |
| ENSG00000139269 | INHBE | inhibin subunit beta E | -1.9232 | 0.0001 |
| ENSG00000176046 | NUPR1 | nuclear protein 1, transcriptional regulator | -1.8809 | 0.0000 |
| ENSG00000111962 | UST | uronyl 2-sulfotransferase | -1.8079 | 0.0000 |
| ENSG00000169429 | CXCL8 | C-X-C motif chemokine ligand 8 | -1.7883 | 0.0000 |
| ENSG00000073756 | PTGS2 | prostaglandin-endoperoxide synthase 2 | -1.7747 | 0.0000 |
| ENSG00000138100 | TRIM54 | tripartite motif containing 54 | -1.7150 | 0.0000 |
| ENSG00000257732 | AC089983.1 | novel transcript, antisense to TXNRD1 | -1.6903 | 0.0000 |
| ENSG00000106034 | CPED1 | cadherin like and PC-esterase domain containing 1 | -1.6553 | 0.0000 |
| ENSG00000108405 | P2RX1 | purinergic receptor P2X 1 | -1.6531 | 0.0000 |
| ENSG00000228626 | AC245100.3 | novel pseudogene | -1.6478 | 0.0000 |
| ENSG00000060566 | CREB3L3 | cAMP responsive element binding protein 3 like 3 | -1.6356 | 0.0000 |
| ENSG00000136244 | IL6 | interleukin 6 | -1.6152 | 0.0000 |

Table S4 The all of 31 significantly up-regulated proteins (Fold Change >2 fold) in MSC group.

| **Protein _ID** | **Protein description** | **Gene name** | **Fold Change** | **Pvalue** |
| --- | --- | --- | --- | --- |
| G3UXV4 | Lymphocyte antigen 6 complex locus protein G6c | Ly6g6c | 6.0577 | 0.0133 |
| P07744 | Keratin, type II cytoskeletal 4 | Krt4 | 4.3102 | 0.0284 |
| C1KG51 | Truncated profilaggrin/filaggrin flaky tail mutant form | Flg | 4.0975 | 0.0330 |
| Q9D7P9 | Serpin B12 | Serpinb12 | 3.9708 | 0.0389 |
| O89094 | Caspase-14 | Casp14 | 3.7697 | 0.0113 |
| P0DP59 | Secreted Ly-6/uPAR domain-containing protein 2 | Slurp2 | 3.7180 | 0.0403 |
| Q148R4 | Serine peptidase inhibitor, Kazal type 5 | Spink5 | 3.2353 | 0.0198 |
| P03958 | Adenosine deaminase | Ada | 3.1970 | 0.0146 |
| E9QNP3 | Hornerin | Hrnr | 3.1522 | 0.0014 |
| Q8VE18 | Protein SMG8 | Smg8 | 3.1088 | 0.0000 |
| O09116 | Small proline-rich protein 3 | Sprr3 | 3.0443 | 0.0330 |
| P18165 | Loricrin | Loricrin | 3.0219 | 0.0367 |
| Q08189 | Protein-glutamine gamma-glutamyltransferase E | Tgm3 | 3.0212 | 0.0217 |
| E9Q4X2 | UDP-glucose glycoprotein glucosyltransferase 2 | Uggt2 | 2.7130 | 0.0000 |
| A0A0B6VJJ9 | Kallikrein related-peptidase 10 | Klk10 | 2.6394 | 0.0289 |
| P35459 | Lymphocyte antigen 6D | Ly6d | 2.6000 | 0.0081 |
| B9EID7 | Suprabasin | Sbsn | 2.5389 | 0.0003 |
| Q99NB5 | Gasdermin-C | Gsdmc | 2.4923 | 0.0024 |
| Q91YK8 | Ly6/PLAUR domain-containing protein 3 | Lypd3 | 2.3897 | 0.0224 |
| P04104 | Keratin, type II cytoskeletal 1 | Krt1 | 2.3619 | 0.0439 |
| Q8BJ03 | Cytochrome c oxidase assembly protein COX15 homolog | Cox15 | 2.3506 | 0.0001 |
| E9Q3E1 | Aldehyde dehydrogenase family 3 member B2 | Aldh3b2 | 2.3353 | 0.0359 |
| Q8CGR6 | Glandular kallikrein KLK13 | Klk13 | 2.3223 | 0.0087 |
| P56501 | Mitochondrial uncoupling protein 3 | Ucp3 | 2.3205 | 0.0047 |
| A0JLQ7 | Pcid2 protein (Fragment) | Pcid2 | 2.2948 | 0.0044 |
| Q499L1 | Metallothionein (Fragment) | Mt4 | 2.2199 | 0.0376 |
| Q05B38 | Krt6b protein (Fragment) | Krt6b | 2.1978 | 0.0480 |
| Q6ZPJ3 | (E3-independent) E2 ubiquitin-conjugating enzyme UBE2O | Ube2o | 2.1930 | 0.0204 |
| Q2VPR3 | 2500003M10Rik protein (Fragment) | Chtop | 2.0677 | 0.0108 |
| Q8VDY7 | Ear11 protein | Rnase2a | 2.0398 | 0.0021 |
| Q8R2Q0 | Tripartite motif-containing protein 29 | Trim29 | 2.0102 | 0.0398 |

Table S5 The all of 12 significantly down-regulated proteins (Fold Change >2 fold) in MSC group.

| **Protein _ID** | **Protein description** | **Gene name** | **Fold Change** | **Pvalue** |
| --- | --- | --- | --- | --- |
| E9PYX3 | Non-specific serine/threonine protein kinase | Mark2 | 0.1512 | 0.0022 |
| Z4YNA3 | Cohesin loading complex subunit SCC4 homolog | Mau2 | 0.3023 | 0.0094 |
| A2A5N3 | Polyadenylate-binding protein | Pabpc1l | 0.3133 | 0.0245 |
| Q9R166 | Zinc finger protein 109 | Zfp109 | 0.3282 | 0.0096 |
| Q3TDL0 | PCI domain-containing protein | Cops7b | 0.3643 | 0.0000 |
| Q99LI9 | Polyribonucleotide 5-hydroxyl-kinase Clp1 | Clp1 | 0.3825 | 0.0000 |
| Q80YV4 | 4-phosphopantetheine phosphatase | Pank4 | 0.4455 | 0.0000 |
| Q9D7T1 | Rab15 effector protein | Rep15 | 0.4667 | 0.0209 |
| Q61733 | 28S ribosomal protein S31, mitochondrial | Mrps31 | 0.4811 | 0.0003 |
| E9PWZ2 | ABPA20 | Scgb1b20 | 0.4833 | 0.0371 |
| E9PYH6 | Histone-lysine N-methyltransferase SETD1A | Setd1a | 0.4930 | 0.0037 |
| Z4YLR9 | Calcium-binding mitochondrial carrier protein SCaMC-2 (Fragment) | Slc25a25 | 0.4941 | 0.0141 |
